# Nuclear up regulation of the BRCA1-associated ubiquitinase BAP1 is associated with tumor aggressiveness in prostate cancers lacking the TMPRSS2:ERG fusion

**DOI:** 10.18632/oncotarget.27270

**Published:** 2019-12-24

**Authors:** Stefan Steurer, Lara Schwemmer, Claudia Hube-Magg, Franziska Büscheck, Doris Höflmayer, Maria Christina Tsourlakis, Till S. Clauditz, Andreas M. Luebke, Ronald Simon, Guido Sauter, Jakob Izbicki, Cornelia Schroeder, Thorsten Schlomm, Hartwig Huland, Hans Heinzer, Alexander Haese, Markus Graefen, Cosima Göbel, Sören Weidemann, Patrick Lebok, David Dum, Christoph Fraune, Sarah Minner, Jan Meiners

**Affiliations:** ^1^Institute of Pathology, University Medical Center Hamburg-Eppendorf, 20246 Hamburg, Germany; ^2^General, Visceral and Thoracic Surgery Department and Clinic, University Medical Center Hamburg-Eppendorf, 20246 Hamburg, Germany; ^3^Martini-Clinic, Prostate Cancer Center, University Medical Center Hamburg-Eppendorf, 20246 Hamburg, Germany; ^4^Department of Urology, Charité - Universitätsmedizin Berlin, 10117 Berlin, Germany

**Keywords:** BAP1, prostate cancer, prognosis, immunohistochemistry

## Abstract

Loss of the putative tumor suppressor BAP1 is a candidate biomarker for adverse prognosis in many cancer types, but conversely for improved survival in others. Studies on the expression and prognostic role of BAP1 in prostate cancer are currently lacking. We used a tissue microarray of 17,747 individual prostate cancer samples linked with comprehensive pathological, clinical and molecular data and studied the immunohistochemical expression of BAP1. BAP1 expression was typically up regulated in cancers as compared to adjacent normal prostatic glands. In 15,857 cancers, BAP1 staining was weak in 3.3%, moderate in 41.6% and strong in 17.4%. Strong BAP1 staining was associated with advanced tumor stage (p<0.0001), high classical and quantitative Gleason grade (p<0.0001), lymph node metastasis (p<0.0001), a positive surgical margin (p=0.0019) and early biochemical recurrence (p<0.0001). BAP1 expression was linked to ERG-fusion type cancers, with strong BAP1 staining in 12% of ERG-negative, but 30% of ERG-positive cancers (p<0.0001). Subset analyses in 5,415 cancers with and 4,217 cancers without *TMPRSS2:ERG* fusion revealed that these associations with tumor phenotype and patient outcome were largely driven by the subset of ERG-negative tumors. Multivariate analysis revealed that the prognostic impact was independent of established prognostic features in ERG negative p<0.001) but not in ERG positive cancers. BAP1 expression was further linked to androgen receptor (AR) expression: Only 2% of AR-negative, but 33% of strongly AR expressing cancers had strong BAP1 expression (p<0.0001). In conclusion, this study shows that BAP1 up regulation is linked to prostate cancer progression and aggressiveness.

## INTRODUCTION

Prostate cancer (PCa) is the most diagnosed cancer among males in Western societies [1]. At this point, established prognosticators include histological analysis of biopsies to determine Gleason score and tumor extent, prostate-specific antigen (PSA) and clinical stage. However, current diagnostic analysis still proves prone to inaccuracies that could be reduced by finding a reliable and clinically applicable molecular marker. This could spare patients with otherwise harmless tumors the negative effects of treatment (e.g. incontinence and erectile dysfunction), and identify those patients with aggressive disease for whom the benefits of treatment outweigh its harm [2].

BRCA-1-associated Protein 1 (BAP1) is a nuclear deubiquitinase targeting histone modifying protein complexes that was originally named after its interaction with the E3 ubiquitin-protein ligase breast-cancer type 1 susceptibility protein (BRCA1) [3, 4]. Subsequent research revealed that BAP1 regulates many cellular pathways that are relevant for cell cycle control, cellular differentiation, gluconeogenesis, DNA damage response and apoptosis [5–7]. Al-though the mechanisms of action of BAP1 are still not fully understood, it is believed that one important function is the regulation of transcriptional silencing at the sites of DNA double-strand breakage repair [6]. BAP1 has long been considered a tumor suppressor. Mutation, genomic deletion of its locus at 3p21 or loss of BAP1 expression has been reported from various tumor types such as non-small cell lung cancer [8, 9], renal cell carcinoma [10–12], gall bladder cancer [13], mesothelioma [14, 15] and uveal melanoma [16–18], and has been linked to poor prognosis in most of them [13, 17, 19]. In addition, inactivating germline mutations result in the BAP1 tumor predisposition syndrome, associated with a high risk of tumor development [20, 21]. However, the tumor-associated functions of BAP1 may be more complex than previously thought as some studies suggest a cancer-promoting role. For example, BAP1 loss or germline mutations have been linked to prolonged survival in malignant pleural mesothelioma [11, 12, 22, 23], and BAP1 overexpression appears to promote basal type breast cancers [24] and myeloid neoplasms harboring certain ATRX mutations [25]. Also, a recent meta-analysis of 26 BAP1 expression studies in 10 different cancer types concludes that the prognostic implication of BAP1 alterations depends on the tumor type [26]. Little is known about alterations of BAP1 in PCa. One study reported lack of BAP1 mutations in 45 prostate tumors [27], but data on BAP1 protein expression or its prognostic significance in this disease are currently acking.

To study the clinical impact of BAP1, we immunohistochemically analyzed more than 17.000 PCa, which have been assembled on a tissue microarray during the last 10 years.

## RESULTS

### Technical issues

89.4 % of 17,747 tumor samples were interpretable. The 10.6% of non-informative cases had no tissue sample or insufficient unequivocal cancer tissue in the TMA spot.

### BAP1 expression in normal and cancerous prostate tissue

**Figure 1:**
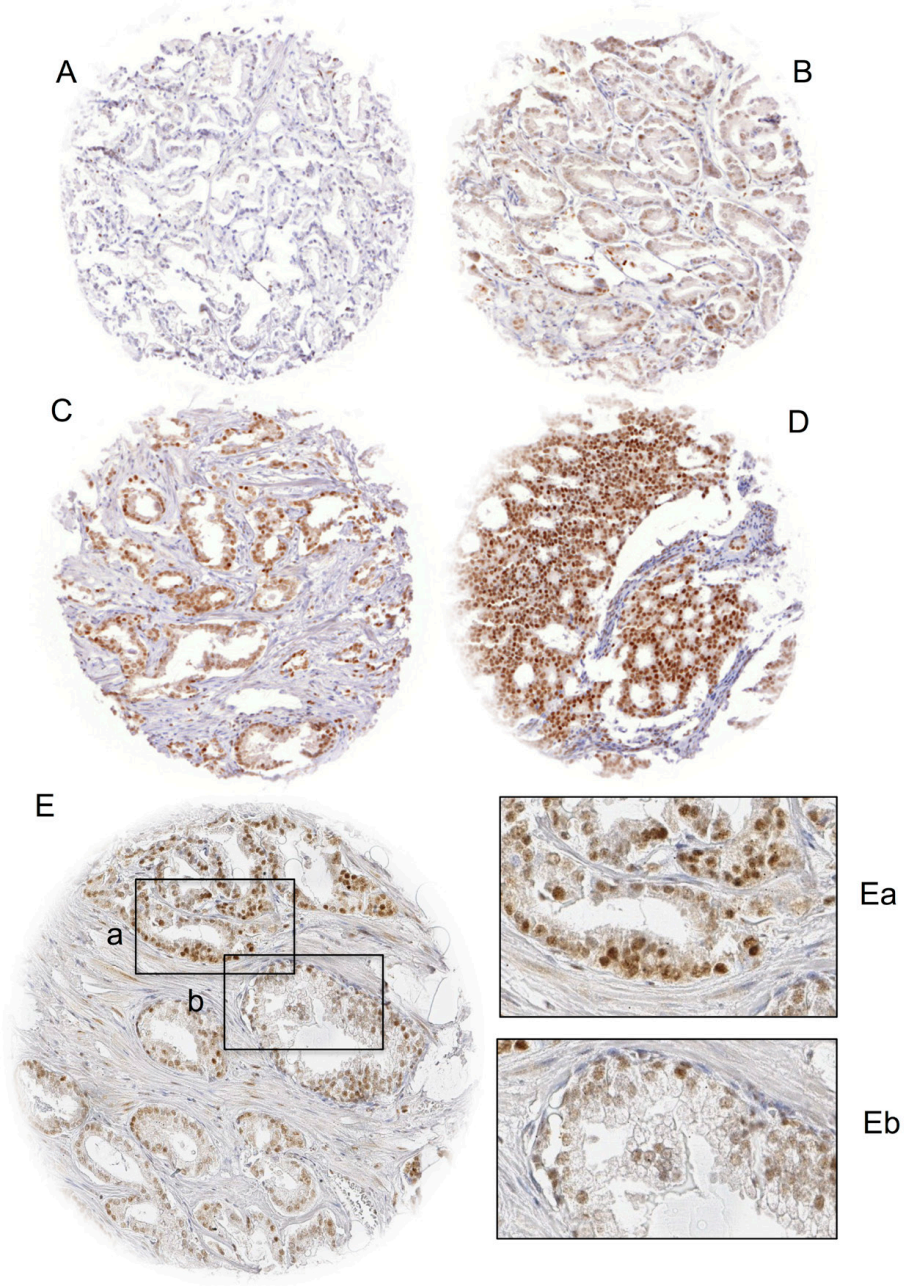
Examples of **(A)** negative, **(B)** weak, **(C)** moderate and **(D)** strong BAP1 staining in prostate cancer and **(E)** BAP1 staining of cancerous **(Ea)** and normal **(Eb)** prostate glands in the same TMA spot. Spot size is 0.6 mm at 100x (inset 400x) magnification.

In order to estimate BAP1 expression in normal prostate glands, we studied several spots containing normal tissue. We found that BAP1 staining ranged from negative to moderate in luminal and in basal cells. In PCa, nuclear staining was seen in 62.3% of 15,857 interpretable tumors. It was considered weak in 3.3%, moderate in 41.6% and strong in 17.4% of PCa. Tissue spots with normal and cancerous glands usually showed higher BAP1 levels in the tumor cells than in normal glands, although there were also rare cases with lower relative BAP1 levels in the cancer cells. Tumors with negative findings typically also lacked BAP1 staining in the adjacent normal tissues. Representative images of nuclear BAP1 staining are shown in Figure 1.

### BAP1 expression and TMPRSS2:ERG fusion status

**Figure 2:**
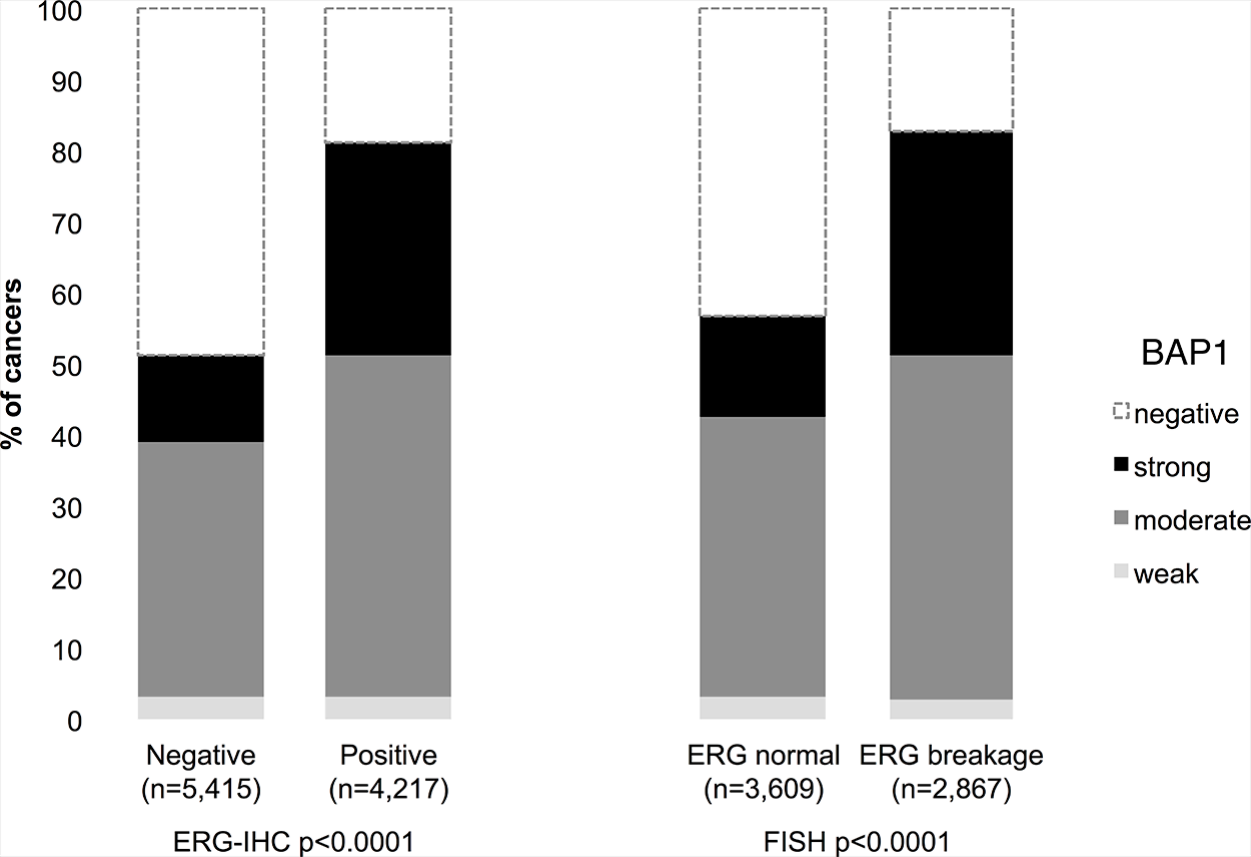
Association between BAP1 staining intensity and ERG status defined by immunohistochemistry (IHC) and fluorescence *in-situ* hybridization (FISH) analysis.

BAP1 staining results were compared with TMPRSS2:ERG data obtained by FISH from 6,476 and by immunohistochemistry from 9,632 tumors. Both, ERG FISH and IHC data were available from 5,365 of these cancers, and concordant results were found in 95.8% cancers. Nuclear BAP1 expression was associated withTMPRSS2:ERG rearrangement and ERG expression: Strong BAP1 positivity increased from 12-14% in 5,415 ERG-negative cancers (by IHC or FISH) to 30-32% in 4,217 ERG-positive cancers (p<0.0001 each, Figure 2).

### BAP1 expression and tumor phenotype

Strong BAP1 staining was associated with adverse tumor features, including advanced tumor stage, high Gleason grade, presence of lymph node metastasis (p<0.0001 each) and a positive surgical margin (p=0.0019, Table 1). Because of the strong link between BAP1 overexpression and ERG rearrangement, the analysis was repeated in the subsets of ERG-negative and ERG-positive cancers. It showed that all associations were solely driven by the subset of ERG-negative cancers (Table 2), while BAP1 staining was unrelated to the analyzed features in ERG-positive cancers (Table 3).

**Table 1:** Association between BAP1 immunostaining and prostate cancer phenotype

			BAP1 (%)			
Parameter	N	Negative	Weak	Moderate	Strong	P
**All cancers**	15 857	37.7	3.3	41.6	17.4	
						
**Tumor stage**						<0.0001
pT2	10 166	41.2	3.0	39.2	16.6	
pT3a	3 508	32.9	3.9	44.2	19.0	
pT3b-pT4	2 119	28.7	3.6	48.5	19.3	
						
**Gleason grade**						<0.0001
≤3+3	3 041	43.7	2.7	38.2	15.4	
3+4	8 394	38.3	3.3	40.1	18.2	
3+4 Tert.5	732	39.1	3.0	42.2	15.7	
4+3	1 543	30.6	4.0	47.4	18.0	
4+3 Tert.5	1 096	30.3	3.2	48.1	18.4	
≥4+4	910	31.6	4.0	47.7	16.7	
						
**Lymph node metastasis**						<0.0001
N0	9 573	36.2	3.5	42.1	18.1	
N+	1 162	29.3	3.4	49.1	18.1	
						
**Preoperative PSA level (ng/ml)**						<0.0001
<4	1 929	32.6	2.6	44.0	20.8	
4-10	9 357	37.7	3.1	41.3	17.9	
10-20	3 330	39.6	3.5	41.1	15.8	
>20	1 140	41.2	5.0	40.7	13.1	
						
**Surgical margin**						0.0019
Negative	12 697	38.4	3.2	41.2	17.2	
Positive	3 104	34.8	3.7	43.2	18.2	

**Table 2:** BAP1 immunostaining and prostate cancer phenotype in ERG *negative* cancers

			BAP1 (%)			
Parameter	N	Negative	Weak	Moderate	Strong	P
**All cancers**	5 415	48.7	3.3	35.8	12.2	
						
**Tumor stage**						<0.0001
pT2	3 643	52.1	2.8	33.8	11.2	
pT3a	1 079	45.8	5.1	36.6	12.5	
pT3b-pT4	677	34.9	3.1	44.9	17.1	
						
**Gleason grade**						<0.0001
≤3+3	1 090	58.3	2.8	29.2	9.7	
3+4	2 879	50.2	3.3	34.9	11.6	
3+4 Tert.5	243	44.9	1.6	40.7	12.8	
4+3	566	39.9	4.4	41.9	13.8	
4+3 Tert.5	328	36.3	4.0	43.0	16.8	
≥4+4	305	33.8	3.9	43.9	18.4	
						
**Lymph node metastasis**						<0.0001
N0	3 149	46.0	3.7	37.2	13.1	
N+	310	31.9	2.9	46.5	18.7	
						
**Preoperative PSA level (ng/ml)**						0.0482
<4	578	44.8	2.9	38.1	14.2	
4-10	3 213	48.8	3.0	35.9	12.3	
10-20	1 167	50.1	3.3	35.1	11.4	
>20	430	48.8	6.3	33.0	11.9	
						
**Surgical margin**						0.0908
Negative	4 341	49.4	3.3	35.5	11.8	
Positive	1 060	45.6	3.5	37.0	14.0	

**Table 3:** BAP1 immunostaining and prostate cancer phenotype in ERG *positive* cancers

			BAP1 (%)			
Parameter	N	Negative	Weak	Moderate	Strong	P
**All cancers**	4217	18.8	3.1	48.1	30.0	
						
**Tumor stage**						0.0065
pT2	2 508	20.7	2.8	47.0	29.4	
pT3a	1 111	16.7	3.4	49.6	30.3	
pT3b-pT4	580	14.5	3.8	50.0	31.7	
						
**Gleason grade**						0.0729
≤3+3	862	21.9	2.9	49.8	25.4	
3+4	2 412	18.6	3.1	46.8	31.5	
3+4 Tert.5	128	21.9	3.1	45.3	29.7	
4+3	409	14.9	4.2	52.1	28.9	
4+3 Tert.5	232	16.4	3.0	47.8	32.8	
≥4+4	171	17.0	2.3	51.5	29.2	
						
**Lymph node metastasis**						0.2321
N0	2 413	17.3	3.4	47.5	31.9	
N+	271	19.2	3.7	51.3	25.8	
						
**Preoperative PSA level (ng/ml)**						0.4984
<4	582	17.0	2.9	49.5	30.6	
4-10	2 587	19.1	3.2	47.3	30.4	
10-20	763	18.7	2.9	48.1	30.3	
>20	252	20.6	3.6	52.8	23.0	
						
**Surgical margin**						0.0639
Negative	3 314	19.6	3.1	47.7	29.5	
Positive	884	15.7	3.1	49.7	31.6	

### BAP1 expression and tumor cell proliferation

Presence of BAP1 staining was linked to increased proliferation as determined by the Ki67 labeling index (Table 4). This association was independent of the Gleason grade as it was observed across subsets with identical Gleason score (≤3+3, 3+4, 3+4 tertiary 5, 4+3, ≥4+4 p<0.0001 each and 4+3 tert. 5; p≤0.0057). Again, subset analyses demonstrated that these associations were driven from the ERG negative subset (p≤0.0007 each).

**Table 4:** Association between BAP1 immunostaining and Ki67 labeling index in Gleason categories and ERG-fusion subsets

Gleason	BAP1	All cancers					ERG-fusion negative					ERG-fusion positive				
		n	Ki67LI^*^			*P*	n	Ki67LI			*P*	n	Ki67LI			*P*
All	Negative	2 243	2.0	±	0.06	*<0.0001*	1 706	1.85	±	0.07	*<0.0001*	499	2.39	±	0.12	*<0.0001*
	Weak	226	2.9	±	0.18		131	3.0	±	0.24		92	2.72	±	0.27	
	Moderate	2 613	3.13	±	0.05		1 196	3.38	±	0.08		1 365	2.93	±	0.07	
	Strong	1 265	3.47	±	0.07		386	3.86	±	0.14		849	3.33	±	0.09	
≤3+3	Negative	591	1.64	±	0.08	*<0.0001*	442	1.51	±	0.1	*<0.0001*	130	2.08	±	0.17	*0.0015*
	Weak	39	3.0	±	0.33		17	2.94	±	0.52		20	3.0	±	0.44	
	Moderate	503	2.4	±	0.09		179	2.69	±	0.16		307	2.27	±	0.11	
	Strong	229	2.93	±	0.14		57	3.33	±	0.28		162	2.86	±	0.15	
3+4	Negative	1 221	2.0	±	0.07	*<0.0001*	922	1.84	±	0.07	*<0.0001*	284	2.32	±	0.14	*<0.0001*
	Weak	122	2.6	±	0.21		70	2.61	±	0.27		52	2.58	±	0.32	
	Moderate	1 442	2.89	±	0.06		639	2.92	±	0.09		781	2.87	±	0.08	
	Strong	749	3.32	±	0.08		196	3.34	±	0.16		539	3.32	±	0.1	
3+4 Tertiary 5	Negative	99	2.33	±	0.26	*<0.0001*	78	2.21	±	0.28	*<0.0001*	21	2.81	±	0.57	*0.1641*
	Weak	6	5.33	±	1.05		3	7.67	±	1.43		3	3.0	±	1.51	
	Moderate	100	3.74	±	0.26		64	3.42	±	0.31		36	4.31	±	0.44	
	Strong	50	3.76	±	0.36		22	4.64	±	0.53		25	3.2	±	0.52	
4+3	Negative	180	2.47	±	0.25	*<0.0001*	139	2.34	±	0.3	*<0.0001*	38	3.0	±	0.46	*0.8606*
	Weak	37	2.84	±	0.54		24	2.92	±	0.73		12	2.83	±	0.81	
	Moderate	284	3.84	±	0.2		152	4.3	±	0.29		126	3.25	±	0.25	
	Strong	102	3.67	±	0.33		43	4.05	±	0.54		59	3.39	±	0.37	
4+3 Tertiary 5	Negative	93	2.7	±	0.38	*0.0057*	77	2.49	±	0.43	*0.0007*	15	3.87	±	0.91	*0.7141*
	Weak	9	3.22	±	1.24		7	3.57	±	1.43		2	2.0	±	2.5	
	Moderate	147	4.15	±	0.31		76	4.72	±	0.44		67	3.46	±	0.43	
	Strong	73	4.56	±	0.43		33	5.15	±	0.66		38	4.13	±	0.57	
≥4+4	Negative	58	2.21	±	0.61	*<0.0001*	48	2.0	±	0.57	*<0.0001*	10	3.1	±	1.89	*0.6276*
	Weak	13	4.15	±	1.29		10	4.5	±	1.25		3	3.0	±	3.44	
	Moderate	135	5.39	±	0.4		84	5.42	±	0.43		48	5.44	±	0.86	
	Strong	61	5.57	±	0.59		35	5.69	±	0.67		25	5.48	±	1.19	

^*^ Mean ± standard error of the mean

### BAP1 and androgen receptor (AR) expression

**Figure 3:**
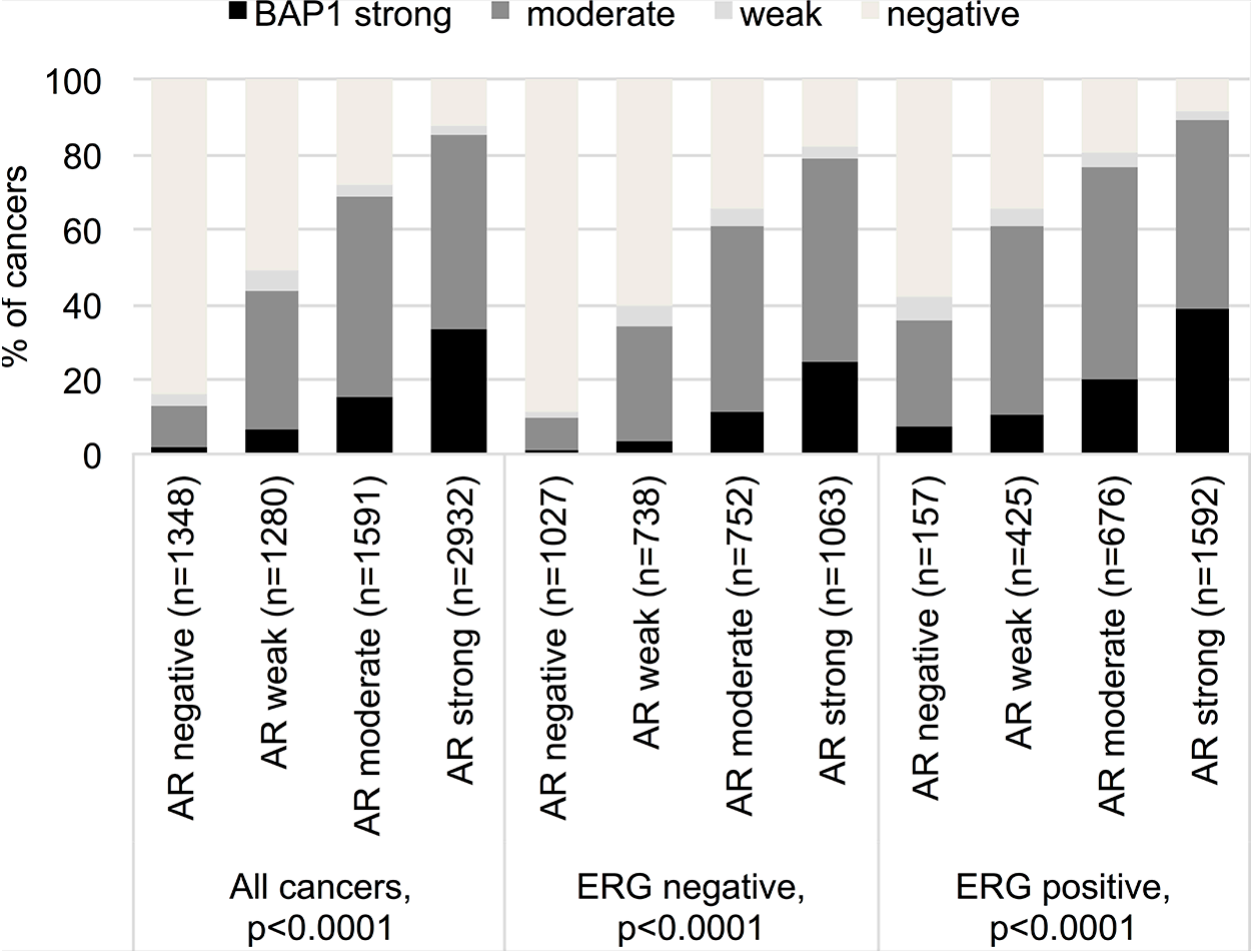
Correlation between BAP1 staining and androgen receptor expression in all cancers, the ERG expression negative and positive subset (IHC).

Data on BAP1 and AR were available from 7,151 cancers [28]. AR expression was associated with nuclear BAP1 staining. Only 2% of AR-negative, but 33% of strongly AR expressing cancers had strong BAP1 expression (p<0.0001). This association held true regardless of the ERG fusion status (p<0.0001 each; Figure 3).

### BAP1 expression and PSA recurrence

**Figure 4:**
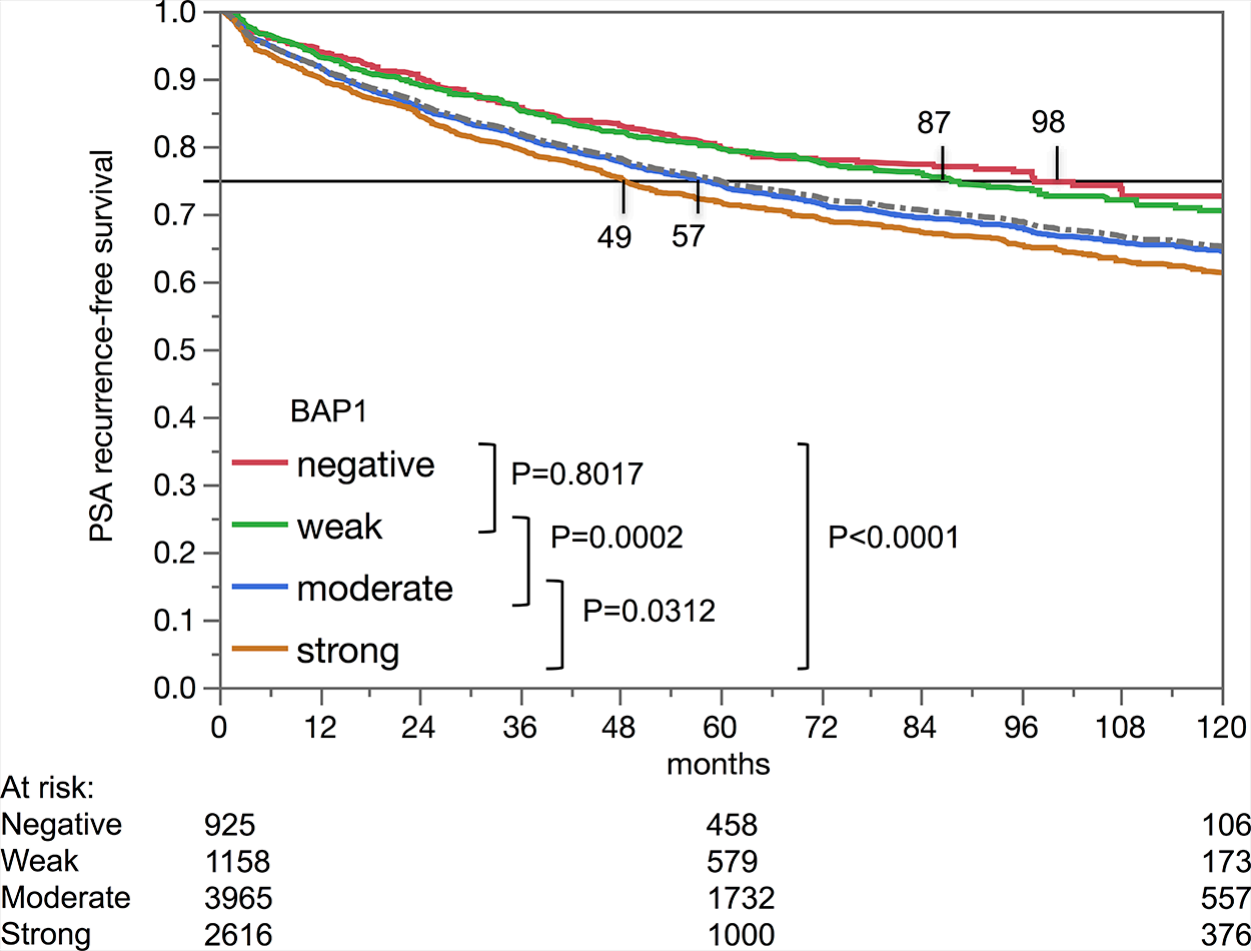
Kaplan-Meier analysis of PSA recurrence-free survival after prostatectomy and BAP1 staining.

**Figure 5:**
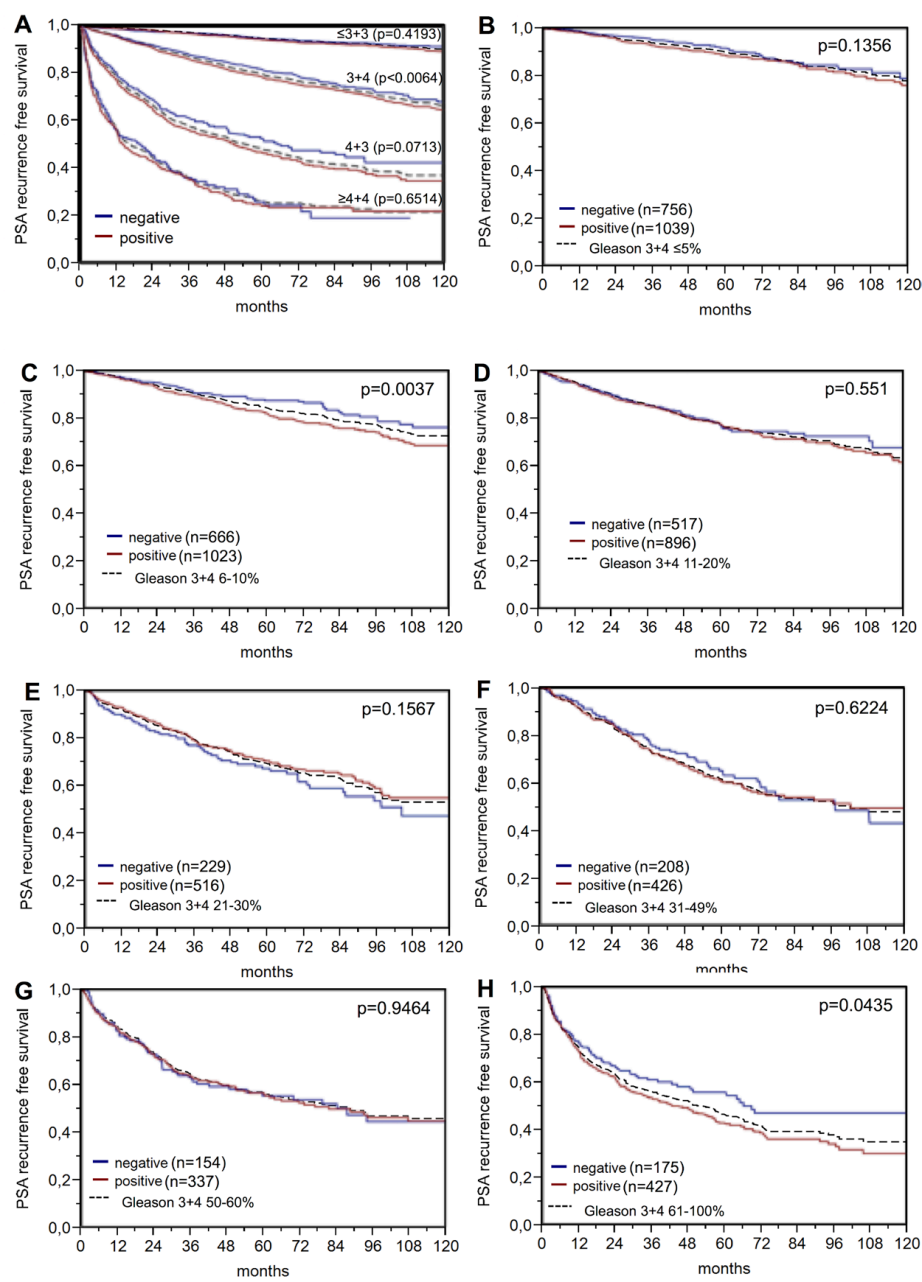
PSA recurrence-free survival after prostatectomy and BAP1 negative versus positive expression in subsets of the ERG expression negative cohort defined by **(A)** the classical Gleason score categories and **(B–H)** the quantitative Gleason score grades defined by the percentage of **(B)** ≤5%, **(C)** 6-10%, **(D)** 11-20%, **(E)** 21-30%, **(F)** 31-49 %, **(G)** 50-60%, and **(H)** ≥61% Gleason 4 patterns. Dashed line shows the combined result of the respective Gleason category for reference.

Nuclear BAP1 staining was linked to early biochemical recurrence (p<0.0001, Figure 4). ERG subset analysis revealed, that the prognostic impact of BAP1 expression was contributed by the ERG negative subset (p<0.0001). BAP1 expression was unrelated to patient outcome in the ERG-positive subset (p=0.1248). A further analysis in the ERG-negative subset revealed that, for subgroups with identical classical and quantitative Gleason grades, BAP1 expression only had a prognostic impact for Gleason 3+4 carcinomas (p=0,006; Figure 5).

### Multivariate analysis

Four different scenarios were tested (Table 5). Scenarios 1 and 2 evaluated postoperatively available parameters (stage, with/without lymph node status (pN), margin status, preoperative PSA value and pathological Gleason grade). Scenario 3 was a mixed model of post- and preoperatively available parameters, while in scenario 4 the preoperative parameters were combined (Gleason grade obtained on the original biopsy, preoperative PSA, cT stage and BAP1 expression). BAP1 expression was an independent prognosticator in PCa (p<0.02) and in the ERG-negative subset (p<0.005 each).

**Table 5:** Hazard ratios (95% confidence intervals) for biochemical relapse after prostatectomy of established risk factors and BAP1 expression in prostate cancer, the ERG negative and positive subsets

Subset	Model		Scenario 4	Scenario 3	Scenario 2	Scenario 1
	Variable	Analyzable (N)	8,171	8,512	8,628	5,450
Total	Gleason grade biopsy	≥4+4 vs. ≤3+3	4.20 (3.69-4.77) ^***^			
	cT stage	T2c vs. T1c	1.92 (1.54-2.38) ^***^	1.70 (1.37-2.11) ^***^		
	Preoperative PSA level	≥20 vs. <4	3.04 (2.49-4.46) ^***^	2.85 (2.36-3.44) ^***^	1.98 (1.65-2.39) ^***^	1.80 (1.46-2.22) ^***^
	**BAP1 expression**	Strong vs. negative	**1.49** (1.27-1.77) ^***^	**1.38** (1.17-1.63) ^***^	**1.32** (1.13-1.56) ^**^	**1.45** (1.21-1.76) ^***^
	Gleason grade prostatectomy	≥4+4 vs. ≤3+3		13.0 (10.6-15.8) ^***^	6.56 (5.32-8.10) ^***^	5.57 (4.25-7.29) ^***^
	pT stage	T4 vs. T2			3.06 (2.71-3.46) ^***^	2.77 (2.40-3.21) ^***^
	Resection margin status	R1 vs. R0			1.39 (1.27-1.53) ^***^	1.25 (1.12-1.39) ^***^
	Nodal stage	N+ vs. N0				1.46 (1.27-1.67) ^***^
ERG neg.		Analyzable (N)	3,962	4,104	4,144	2,681
	BAP1 expression	Strong vs. negative	1.71 (1.38-2.12) ^***^	1.53 (1.24-1.88) ^***^	1.45 (1.18-1.79) ^**^	1.63 (1.28-2.08) ^***^
ERG pos.		Analyzable (N)	3,194	3,355	3,414	2,192
	BAP1 expression	Strong vs. negative	1.44 (0.92-2.45)	1.47 (0.94-2.50)	1.29 (0.81-2.08)	1.24 (0.77-2.14)

Scenario 4 combines preoperatively available parameter (Gleason score obtained on the original biopsy, clinical tumor (cT) stage, and PSA level) with the postoperative BAP1 expression. In scenario 3 the Gleason at biopsy is replaced by the Gleason obtained on radical prostatectomy. In scenario 2, cT-stage is superseded by pathological tumor (pT) stage and surgical margin (R) status. In scenario 1 the lymph node (pN) stage is added. ^*^ p≤0.05, ^**^ p≤0.001, ^***^ p≤0.0001

## DISCUSSION

In this study we show that nuclear BAP1 expression is an independent predictor of poor prognosis in ERG negative PCa.

Nuclear BAP1 staining was seen in 62% of PCa, including 17,4% tumors with strong BAP1 staining intensity. Normal prostatic epithelial tissue showed variable but generally lower BAP1 expression levels ranging between negative and moderate positive staining. That BAP1 staining intensities were often higher in cancer cells than in adjacent normal prostate glands suggests that BAP1 usually becomes overexpressed during tumor development. Comparable studies on BAP1 in prostate tissues are currently lacking in the published literature. However, the human protein atlas (https://www.proteinatlas.org/ENSG00000163930-BAP1/tissue/prostate) shows examples of BAP1 staining that are in line with our findings, including six samples of normal prostate glands (with low to medium intensity staining) and 23 samples of PCa with variable levels of positivity ranging from negative to strong using two different anti-BAP1 antibodies including HPA028814, which was used in our study [29].

A strong association between BAP1 up regulation, adverse tumor phenotype and clinical outcome was found in our cohort of more than 15.800 patients. Similar findings have been reported from malignant pleural mesotheliomas, where BAP1 overexpression was also linked to aggressive tumor features or shortened survival [15, 30–33]. These observations are in contrast to data described for most other tumor types that have been analyzed for BAP1 alterations so far. Reduced BAP1 expression has been linked to poor prognosis and adverse tumor features in renal carcinoma [10–12], colorectal cancer [34], gastric adenocarcinoma [35], non-small cell lung cancer [8, 9], gall bladder cancer [13] and uveal melanoma [16–18, 36]. These data suggest that BAP1 may function differently in different tumor types. Whereas the tumor suppressive role has been attributed to BAP1’s important involvement in DNA double strand breakage repair [37], there is emerging evidence that BAP1 can also promote tumor growth when it is overexpressed in particular molecular environments. For example, target genes of BAP1 deubiquitination include mutant ATRX in myeloid neoplasms [25] and Krüppel-like factor 5 (KLF5) in basal-like breast cancers [24], which both become stabilized by BAP1 and consequently accelerate tumor growth [24, 25]. That BAP1 interacts with KLF5 is of potential interest. KLF5 is a hormone-regulated gene in PCa and may have an oncogenic or tumor suppressive role depending on posttranscriptional modifications [38–40].

Our analysis of molecularly defined tumor subgroups revealed that the prognostic impact of BAP1 was almost entirely driven by the ERG negative subset. About 50 percent of PCa carry *TMPRSS2:ERG* fusions [41, 42] leading to a constitutive overexpression of ERG [28]. ERG overexpression by itself had no prognostic relevance, at least in patients not receiving systemic therapy [43]. However, ERG regulates more than 1,600 genes in prostate epithelial cells. Some proteins are mitigated, others intensified. The substantially higher BAP1 expression in ERG positive (30% with strong BAP1 positivity) than in ERG negative cancers (12% with strong BAP1 positivity) provides strong *in vivo* evidence for an ERG-BAP1 interaction. BAP1 may be directly regulated by ERG, since analysis of the BAP1 promoter/enhancer region using GeneHancer [44] indicates binding sites for 179 transcription factors, including one for ETS transcription factors such as ERG. A functional interaction may also exist through BAP1’s binding partner BRCA1, which contributes to the regulation of WNT-signaling [45]. Activation of Wnt signaling ranks among to the best-known consequences of ERG activation [41, 46, 47], and it can be assumed that most factors involved in this pathway undergo expression changes once ERG becomes active.

That BAP1 expression didn’t change patient outcome in the ERG positive subset argues for circumstances related to the ERG specific cellular microenvironment not only modifying BAP1 expression levels but also impacting its biological effects. This phenomenon has been observed in earlier studies, in which various molecular features were observed that were exclusively prognostic in ERG positive (SOX9, [48]; AZGP1, [49]; HOOK3, [50] or in ERG negative cancers (YB-1, [51], p16, [52], BCAR1, [53]), but not in both groups. As an alternative explanation for the lack of prognostic impact of BAP1 in the ERG positive subset, we cannot rule out that our experimental set-up was more sensitive to expression differences at the lower level (ERG negative subset) than at the higher level (ERG positive subset). Irrespective of the underlying mechanism, the selective prognostic impact of BAP1 in ERG negative cancers demonstrate, that the applicability (and perhaps thresholds) of prognostic markers may depend on individual molecular tumor features. This represents a challenge for the development of biomarkers that, ideally are applicable to every patient.

Other molecular markers with associations to BAP1 up regulation included androgen receptor and the Ki67 cell proliferation marker. The massive increase of BAP1 expression with AR expression strongly suggests a functional interaction. This is supported by one *in vitro* study showing that androgen signaling was among the pathways that become deregulated in a cell line model harboring an inactivating BAP1 mutation [54]. The massive increase of BAP1 expression with tumor cell proliferation was expected, as BAP1 regulates cell proliferation via deubiquitination of its target protein host cell factor-1 (HCF1), which plays a critical role at multiple stages of the cell cycle [55, 56]. That the impact of BAP1 on proliferation was much stronger in ERG negative than in ERG positive cancer further supports the notion that ERG activation may interfere with functions of BAP1.

The results of this study suggest that BAP1 expression may represent a useful marker in ERG negative cancer. In this subset, BAP1 expression had a significant impact, which was independent of established prognostic parameters, irrespective of whether all available features or only preoperatively available prognostic parameters were included into the analysis. It should be noted, however, that its independent prognostic relevance is not the only important criterion for a prognostic feature in PCa. Most established prognostic parameters that are typically included into multivariate analyses in PCa studies are statistically strong but suffer from shortcomings in clinical practice. pT stage and nodal status cannot be determined before the prostate is surgically removed and therefore cannot be used for preoperative therapeutic decision-making. Even in the postoperative situation, the detection rate of lymph node metastasis is highly variable and greatly depends on the extent of surgery and the pathological work-up of the removed tissue [57]. Gleason grade, the most powerful prognostic marker available preoperatively, suffers from substantial interobserver variability, reaching up to 40% in individual biopsies [58]. That BAP1 expression lacks prognostic impact in cancers with identical quantitative Gleason grade demonstrates the statistical power of the quantitative Gleason grading system, however, it is not universally applied and does not solve all issues of interobserver variability in PCa grading.

In summary, up regulation of BAP1 is associated with adverse features, rapid cell proliferation and poor patient prognosis in PCa. BAP1 expression analysis may have prognostic utility either alone or, more likely, in combination with other biomarkers.

## MATERIALS AND METHODS

### Patients

The 17,747 patients had radical prostatectomy at the Department of Urology and the Martini Clinic at the University Medical Center Hamburg-Eppendorf between 1992 and 2015. Specimens were analyzed according to a standard procedure [59]. Classical Gleason categories and “quantitative” Gleason grading was performed as described previously [58]. In brief, for quantitative Gleason grading the percentage of Gleason 4 patterns was recorded and the 3+4 and 4+3 cancers subdivided in subgroups with ≤ 5%, 6-10%, 11-20%, 21-30%, 31-49%, respective 50-60%, 61-80% and > 80% Gleason 4 pattern. Follow-up for the time to PSA recurrence was available for a total of 12,859 patients (median 48 months, range: 1 to 276 months; Table 6). Prostate specific antigen (PSA) levels were measured following surgery and PSA recurrence was defined as a postoperative PSA of at least 0.2 ng/ml and increasing at subsequent measurements. The TMA was manufactured as described earlier in detail [60]. A corresponding TMA database contained prior results on ERG expression, *ERG* break apart FISH analysis [43], Ki67 labeling index (Ki67LI) data [61], androgen receptor (AR) expression [28], and deletion status of 5q21 (*CHD1*) [62], 6q15 (*MAP3K7*) [63], *PTEN* (10q23) [64], 3p13 (*FOXP1*) [65]. The use of anonymized diagnostic leftover tissues was in accordance with local laws (HmbKHG, §12a) and approved by the local ethics committee (Ethics Commission Hamburg, WF-049/09). All work has been carried out in compliance with the Helsinki Declaration.

**Table 6:** Pathological and clinical data of the arrayed prostate cancers

	No. of patients (%)	
	Study cohort on TMA^*^	Biochemical relapse among categories
**Follow-up**		
N	14 464	3 612 (25%)
Mean/median-time to PSA recurrence (month)	56.3/48.0	-
**Age (y)**		
≤50	433	66 (15.2%)
51-59	4 341	839 (19.3%)
60-69	9 977	2 073 (20.8%)
≥70	2 936	634 (21.6%)
**Pretreatment PSA (ng/ml)**		
<4	2 225	313 (14.1%)
4-10	10 520	1 696 (16.1%)
10-20	3 662	1 043 (28.5%)
>20	1 231	545 (44.3%)
**pT stage (AJCC 2002)**		
pT2	11 518	1 212 (10.5%)
pT3a	3 842	1 121 (29.2%)
pT3b	2 233	1 213 (54.3%)
pT4	85	63 (74.1%)
**Gleason grade**		
≤3+3	3 570	264 (7.4%)
3+4	9 336	1 436 (15.4%)
3+4 Tert.5	1 697	165 (9.7%)
4+3	2 903	683 (23.5%)
4+3 Tert.5	1 187	487 (41%)
≥4+4	999	531 (53.2%)
**pN stage**		
pN0	10 636	2 243 (21.1%)
pN+	1 255	700 (55.8%)
**Surgical margin**		
Negative	14 297	2 307
Positive	3 388	1 304

^*^ Numbers do not always add up to 17 747 in the different categories because of cases with missing data. Abbreviation: AJCC, American Joint Committee on Cancer.

### Immunohistochemistry

Freshly cut TMA sections were immunostained in one experiment. Slides were deparaffinized and exposed to 121°C in pH 7.8 Tris-EDTA buffer for 5 minutes. HPA028814 primary antibody specific for BAP1 (rabbit polyclonal antibody, Sigma-Aldrich, St. Louis, Missouri, USA, dilution 1:150) was applied at 37°C for 60 minutes. This antibody was validated by Western blot and protein array in the human protein atlas [29, 66]. Of note, the product was discontinued while manuscript was under review. Bound antibody was visualized with the EnVision Kit (Dako, Glostrup, Denmark) according to the manufacturer’s directions. BAP1 staining was mainly seen in the nucleus and typically paralleled by cytoplasmic staining of lower intensity. Only nuclear staining was scored in this study. As BAP1 typically stained the nucleus in all (100%) tumor cells of a BAP1-positive tissue spot, only the staining intensity was assessed on a four-step scale: negative (0+), weak (1+), moderate (2+) and strong (3+). Scoring was done at 100-200x magnifications by a single pathologist.

### Statistics

Contingency tables were analyzed with the chi^2^-test to search for associations between molecular parameters and tumor phenotype. Kaplan-Meier curves were calculated and the log-rank test was applied to detect differences between groups. Cox proportional hazards regression analysis was performed to test for independence and significance between pathological, molecular and clinical variables. JMP 11 was applied (SAS Institute Inc., NC, USA).
